# Translation, cross-cultural adaptation and measurement proprieties of the German version of the Allodynia Symptom Checklist (ASC-12)

**DOI:** 10.1186/s10194-023-01697-9

**Published:** 2023-12-01

**Authors:** Tetiana Marusich, Tibor M Szikszay, Anne Sennholz, Kerstin Luedtke, Gabriela F. Carvalho

**Affiliations:** 1 Institut für Gesundheitswissenschaften, Studiengang Physiotherapie, Pain and Exercise Research Luebeck (P.E.R.L), Universität zu Lübeck, Lübeck, Deutschland; 2 Department of Physiotherapy, Faculty of Health, Safety and Society, Furtwangen University, Furtwangen, Germany

**Keywords:** Primary headaches, Pain sensitivity, Quantitative sensory testing, Psychometric proprieties

## Abstract

**Background:**

Cutaneous allodynia is highly prevalent among migraineurs and is associated with a poor prognosis. The Allodynia Symptom Checklist (ASC-12) is a comprehensive questionnaire to identify the presence and severity of allodynia. Our aim was to translate and adapt the ASC-12 to German and evaluate its measurement properties.

**Methods:**

Following the COSMIN guidelines, 80 migraine patients were enrolled in the study to evaluate the stages of translation (*n*=30) and measurement propriety assessment (*n*=50), respectively. After reaching a final version, the German ASC-12 was assessed for structural validity, internal consistency, test-retest reliability, construct validity and absolute agreement, using mechanical and thermal pain thresholds as reference method.

**Results:**

The German version of the ASC-12 presented an adequate structural validity compatible with the original version of the questionnaire. Its internal consistency ranged from 0.70 to 0.80 considering the total score and the thermic, static and dynamic mechanic subdomains. The total score presented excellent reliability (ICC: 0.85) with a standard error of measurement of 1.15 points and smallest detectable change of 3.40 points. ASC-12 total scores were correlated with headache intensity (r=0.38, *p*=0.004), headache disability (r=0.37, *p*=0.004) and cold pain thresholds (r=0.28, *p*=0.025). The thermic allodynia ASC-12 scores were correlated with cold (r=0.36, *p*=0.005) and heat (r=-0.30, *p*=0.010) pain thresholds, while the static mechanical allodynia ASC-12 scores correlated with mechanical pain threshold (r=0.29, *p*=0.019) and with mechanical pain sensitivity (r=0.24 to 0.28, *p*< 0.045). Despite no significant bias between methods, quantitative sensory testing (QST) results and ASC-12 scores tend to disagree.

**Conclusion:**

The German version of the ASC-12 is available for research and clinical settings and presented adequate measurement proprieties, as the original version. Despite the correlation between the ASC-12 and QST, one method cannot be replaced by the other.

**Supplementary Information:**

The online version contains supplementary material available at 10.1186/s10194-023-01697-9.

## Key findings

- The German version of the ASC-12 exhibit semantic and idiomatic equivalence to the original English version.
- The German ASC-12 presented adequate internal consistency, structural validity and reliability.
- Total ASC-12 scores correlate with headache intensity, disability and cold pain thresholds
- The QST evaluation cannot be replaced by the ASC-12 in the evaluation of cutaneous allodynia.

## Introduction

Prolonged and/or repeated noxious stimuli can sensitize the nociception pathways and lead to cutaneous allodynia, defined as the perception of pain provoked by an innocuous thermic and/or mechanic stimulus applied over the skin [1, 2]. Up to 63% of patients with migraine present cutaneous allodynia during the attacks [3], but it can also occur interictally [4, 5].

The presence of cutaneous allodynia increases the risk of migraine chronification [1, 6], as it is considered a central sensitization marker [2]. Despite increased prevalence of central sensitization in patients with chronic migraine, it is also often observed among individuals with episodic migraine [1]. Cutaneous allodynia is related to poorer clinical outcomes such as increased depression and anxiety levels, higher migraine disability, lower quality of life, sleep disorders, medication overuse, and greater pain intensity [7–9]. It can predict refractory pharmacological treatment response since triptans are more likely to be effective if their intake precedes the onset of cutaneous allodynia [10–12]. Furthermore, cutaneous allodynia is considered a critical factor for suicidality, along with the presence of osmophobia [13].

The identification of cutaneous allodynia in clinical practice is crucial due to its relevance to the migraine prognosis, development of comorbidities and response to treatment. However, quantitative sensory testing (QST) – considered the gold standard to evaluate cutaneous allodynia [14] – has limited feasibility in the clinical setting due to high costs for equipment, requirement of specialized training and the time needed for testing [15]. The Allodynia 12-item questionnaire (Allodynia Symptom Checklist-12, ASC-12) was developed by Lipton and colleagues to assess the presence and severity of cutaneous allodynia [3]. It is a quick and valid tool that considers thermic, mechanical static and mechanical dynamic dimensions of pain perception [1, 3]. Despite being widely used for both research and clinical settings worldwide, it is not yet translated or adapted to German. Therefore, the aim of this study was to translate and culturally adapt the ASC-12 to German. We further aimed to assess its measurement proprieties, including structural validity, reliability, internal consistency, construct validity and agreement with the QST assessment protocol.

## Materials and methods

### Study design and sample

This study was performed at the University of Luebeck in Germany and approved by the local Ethics Committee (process number: 21-506). The procedure was carried out in two parts with two different samples of patients with migraine. First, the translation and cross-cultural adaptation of the ASC-12 to German was accomplished and tested in 30 patients, according to the Guidelines for the process of cross-cultural adaptation of self-reported measures [16]. Afterwards, the measurement proprieties assessment of the German version of the ASC-12 was performed in 50 patients, according to the COSMIN guidelines [17].

All participants were screened for migraine according to the ID migraine questionnaire [18] and had their diagnosis performed by a neurologist in accordance with ICHD-III criteria [19]. They were enrolled in the study if they were between 18 to 65 years old and had at least one migraine attack within the last month. Patients with concomitant pain syndromes within the last 3 months, concomitant headache diagnosis and/or neurological, cognitive or psychiatric disorders, and headache during the assessment were excluded. Demographic information, medication intake, headache characteristics and headache impact (HIT-6™ GlaxoSmithKline) [20] were informed by all participants.

### Translation and cross-cultural adaptation

The ASC-12 includes 12 questions that encompass everyday activities such as combing the hair, shaving the face, wearing glasses, taking a shower, among others. The patients indicate the frequency of pain or unpleasantness during a migraine attack by rating the options: *does not apply to me*, *never*, *rarely*, *less than half the time*, and *half the time or more*. The response *less than half of the time* is scored as 1 point, and *half the time or more* as 2 points, while the other options do not receive any score. The sum of points equal or greater than 3 indicates the presence of cutaneous allodynia. Scores between 3 and 5 indicate mild, from 6 to 8 moderate, and 9 or greater severe cutaneous allodynia [3].

The translation process and cross-cultural adaptation of the ASC-12 to German was conducted following six stages [16]:

1. English to German translation, performed by two native German speakers fluent in English, who independently translated the checklist into German.
2. Synthesis of the translation to conceive one agreed version of the questionnaire.
3. Back translation, performed by two German translators, whose native language is English.
4. Expert committee review of the translations and consent on a pre-final version of the questionnaire. Eight members composed the expert committee, which consisted of post-graduation students, clinicians, professors in the field, and the translators involved in the previous stages.
5. Application of the pre-final version to 30 patients with migraine to evaluate comprehension difficulties of the questions.
6. Submission of the documents to the expert committee once the final version of the questionnaire was reached.

The last stage of the translation and cross-cultural adaptation was carried out between May and June of 2022 among 30 patients with migraine through the platform limesurvey.org (Table 1). The online survey was disseminated through simple random sampling in the migraine league organization. It included the pre-final version of the ASC-12 questionnaire conceived in stage 4, along with clinical and demographic questions. In addition, participants were asked if they had doubts regarding the meaning of words and understanding of the ASC-12 after its completion. In case of doubts from more than 10% of patients on the same question, a revision and rephrasing of the item(s) is required, with subsequent testing among 30 new patients with migraine [16]. At the beginning of the survey, each participant received an individual identification number through a link to “limesurvey.org” to prevent unauthorized access and multiple participation.

**Table 1:** Sample demographics (mean, SD and percentages) for each study stage

	Translation and cross-cultural adaptation (*n*=30)	Measurement proprieties assessment^a^ (*n*=50)
Age (years)	34.7 (12.8)	27.9 (10.1)
Gender (%, n female)	80% (24)	84% (42)
Migraine onset (years)	17.0 (12.8)	12.3 (8.8)
Presence of aura (%)	57%	56%
Migraine frequency (days/month)	2.6 (2.5)	4.5 (3.9)
low-frequency episodic migraine (1 -7 days, %)	93%	82%
high-frequency episodic migraine (8 - 14 days, %)	7%	14%
Chronic migraine (>15 days, %)	0%	4%
Migraine intensity (NRS, 0-100)	66.0 (17.1)	59.9 (18.7)
Migraine phase (ictal phase)	-	42% (21)
Headache impact test (HIT-6, scores)	-	37.8 (10.6)
Prevalence of allodynia (ASC-12 ≥ 3)	53%	76%
ASC-12 total (scores)	3.2 (2.5)	5.4 (3.2)
ASC-12 thermic (scores)	1.5 (1.9)	3.2 (2.1)
ASC-12 static mechanical (scores)	0.9 (1.1)	2.0 (1.4)
ASC-12 dynamic mechanical (scores)	0.8 (1.2)	0.1 (0.5)

*NRS* Numeric rating scale, *ASC-12* Allodynia symptom checklist, *SD* Standard deviation

^a^Measurement proprieties assessment included assessment of internal consistency, test-retest reliability, absolute agreement, structural and construct validity

### Measurement proprieties evaluation

Consecutive patients with migraine were recruited for an in-person appointment at the University of Luebeck, Germany. This sample was used to evaluate the questionnaire’s internal consistency, test-retest reliability, absolute agreement, structural and construct validity. Further than the collection of the self-reported outcomes previously described, patients underwent a standardized QST protocol established by the German Neuropathic Pain Research Network [21], to determine construct validity and absolute agreement. In order to match the dimensions of the ASC-12, the QST protocol consisted of evaluation of cold and heat pain thresholds, mechanical pain thresholds and mechanical pain sensitivity. To also determine reliability, the participants received one week later [22, 23] a *limesurvey* link via email to fill out again the final version of the German ASC-12.

Thermal pain thresholds were assessed with a thermal contact stimulator (TCS, QST.Lab, (TCS; André Dufour, University of Strasbourg). The area of stimulation of the TCS (T11) has a surface of 9cm^2^. The thermode was used to determine individual heat and cold thresholds starting with an initial temperature of 32°C with increments/decrements of 1°C/second, with a maximal range from 0°C to 60°C. Patients were instructed to press a stop button once the temperature change first elicited pain. The average of three repetitions for each modality was used for data analysis.

Mechanic pain threshold was determined using a set of weighted pinprick stimulators (8, 16, 32, 64, 128, 256 and 512mN, MRC Systems GmbH, Heidelberg, Germany). Five series of ascending and descending stimuli were performed and patients reported the stimulator that elicited a sharp sensation for each of the series. The geometric mean was used for analysis.

Mechanical pain sensitivity was tested using the same set of pinprick stimulators, a soft brush (BR), a cotton wisp (CW), and a Q‐tip (QT) over the skin. The CR, CW and QT were applied with a single stroke at a velocity of 2 to 3 cm/s over approximately 2cm in length of the skin. Each stimulus was applied over the skin in a randomized order and this time subjects were instructed to rate their pain intensity ranging from 0 to 100 using a numeric pain scale (NRS). The degree of pain intensity was defined using the geometric mean of the 5 trials for each type of stimulus.

All measurements were done at the distribution of V1 on the dominant headache side. If there was no dominant headache side reported, the evaluation site was chosen by simple randomization.

### Statistical analysis

After the translation and cross-cultural adaptation phase, a confirmatory factor analysis [24] was used to assess the structural validity and to verify if the questionnaire exhibited the same three factors (thermic, static mechanical and dynamic mechanical allodynia) as the original ASC-12 version. The factor loading of each question with standardized values above 0.3 represents a relevant contribution to a dimension. The goodness-of-fit of the model was confirmed according to the following parameters: Tucker-Lewis-Index (TLI) and comparative fit index (CFI) above 0.9, mean square error root of approximation (RMSEA) and standardized root mean square residual (SRMR) below 0.08 [25]. A path diagram was used to present the standardized coefficients of each question and each dimension.

The test-retest ASC-12 reliability was assessed using two-way random-effects, absolute agreement Intraclass Correlation Coefficient (ICC), based on total scores and scores of each of the subscales in the first and second application of the German version of the ASC-12. ICCs with values below 0.45 indicated poor reliability, between 0.50 and 0.75 satisfactory, and greater than 0.75 excellent reliability [22]. Based on the reliability results, the standard error of measurement (SEM, SEM = SD × √1−ICC) and smallest detectable change on an individual level (SDC, SDC_ind_ = SEM × 1.96 × √2) [25] were calculated. The German version of the ASC-12 was assessed for internal consistency using Cronbach’s alpha coefficient for each dimension and for the total score. Values between 0.70 and 0.95 were considered adequate, as lower values indicate a lack of correlation between the items and high values indicate item redundancy [25].

The construct validity of the ASC-12 was analyzed using a two-tailed Pearson’s correlation test between the ASC-12 and clinical outcomes. The correlation of total scores was evaluated using the HIT-6 questionnaire scores, headache intensity, frequency and onset, mechanical pain sensitivity, mechanical, cold and heat pain thresholds. Correlations between the thermal allodynia scores with cold and heat were also evaluated. Furthermore, correlations between static and dynamic mechanical allodynia scores were performed by testing mechanical pain thresholds and mechanical pain sensitivity. Correlation values below 0.30 were considered weak, between 0.31 and 0.70 moderate, above 0.71 strong correlations [24].

We further tested the absolute agreement between ASC-12 scores with mechanical, cold and heat pain thresholds through Bland-Altman analysis. The mean difference of methods (bias) in relation to the maximum agreement based on normalized scores of outcomes was estimated. Limits of agreements (LoA) were calculated considering the mean difference of both methods ± 1.96 SD [26]. The presence of zero within the 95%CI bias indicates no systematic bias [23]. In addition, the hypothesis test of agreement proposed by Shieh was tested, and the conclusion of method agreement was reached by rejecting the null hypothesis [25].

The confirmatory factor analysis and absolute agreement analysis were performed using the software JAMOVI (version 2.3.21, The Jamovi Project, Sydney, Australia). The SPSS software (version 26.0, IBM, New York, USA) was used to perform the remaining analyses. An alpha level at 5% was considered and there were no missing data.

## Results

### Translation and cross-cultural adaptation

During the cross-cultural adaptation of the ASC-12, some English words and expressions were adapted to improve clarity. The expression “Pulling your hair back (e.g., ponytail) was adapted to “*Zurückhalten der Haare (z. B. Pferdeschwanz)*”. The expression “Resting your face or head on a pillow” was adapted to “*beim Ablegen des Gesichts oder Kopfes auf einem Kissen*”. “Exposure to heat” and “Exposure to cold” were translated to “*bei Hitze*” and “*bei Kälte*”. The classification of allodynia was adapted as follows: “None” as “*keine*”, “Mild” as “*leichte*”, “Moderate” as “*moderate*” and “Severe” as “*schwere*”. After the translation process, a consensus regarding the pre-final version of the questionnaire was reached by the expert committee and translators (Supplemental material).

Two participants (7%) reported not understanding the difference between “does not apply to me”/“*betrifft mich nicht*” and “never”/“*Niemals*”. Since there were no further comprehension doubts in same item by more than 10% (3 participants), the pre-final version was considered the German ASC-12 final version (Supplemental material). Demographic and headache characteristics of the 30 patients included in the translation and cross-cultural adaptation stage of the study can be found in Table 1.

Other comments from participants (10%) included the report of allodynia in other situations such as wearing a face mask, helmets, headphones or earplugs. Participants (13%) also reported that while having a headache they entirely avoided some activities (i.e., as wearing glasses, tight clothes, ponytail or shaving the face), since they anticipated discomfort. In this case, they considered the “does not apply to me”/“*betrifft mich nicht*” as the appropriate answer, since they never experienced discomfort by performing the described activity.

### Measurement Proprieties

The assessment of the ASC-12 measurement proprieties took place between August and December 2022. From 51 recruited patients with migraine, one was excluded due to the concomitant diagnosis of tension-type headache. The demographic and clinical characteristics of the sample recruited in this stage of the study are shown in Table 1.

#### Structural validity

**Fig. 1:**
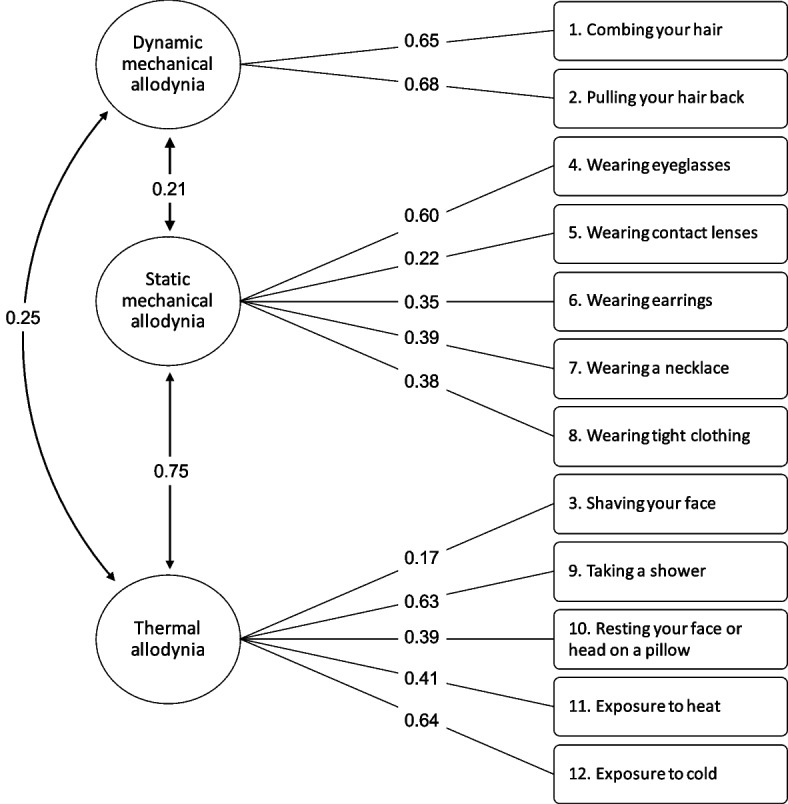
Structure of the German version of the ASC-12 analyzed by the confirmatory factor analysis. The standardized coefficients represent the individual contribution of each question to the model

A confirmatory factor analysis was used to perform the structural validity assessment of the German ASC-12 according to the three pre-defined dimensions of the questionnaire: thermic allodynia, static and dynamic mechanical allodynia. The model-fit was considered adequate as all values of CFI and TLI were very close to the pre-defined thresholds. Furthermore, RMSA and SRMR were equal or below 0.08 (model 1, Table 2). Figure 1 shows the path diagram with the factor loadings of each dimension item. The standardized coefficients ranged between 0.17 and 0.68. The questionnaire items “shaving your face” and “wearing contact lenses” did not contribute significantly to the goodness-of-fit, since they presented factor loadings below 0.30. A second model without these two items was estimated (model 2, Table 2), but no improvement of the model-fit was observed.

**Table 2:** Model-fit of the German version of the Allodynia Symptom Checklist (ASC-12)

	CFI	TLI	RMSEA (90% CI)	SRMR
Model 1	0.88	0.86	0.05 (0.00 to 0.09)	0.08
Model 2^a^	0.80	0.72	0.08 (0.02 to 0.12)	0.08

*CFI* Comparative fix index, *TLI* Tucker Lewis Index, *RMSEA* Root mean square error of approximation, *SRMR* Standardized Root Mean Square Residual

^a^without the items “shaving your face” and “wearing contact lenses”

#### Reliability

The mean ASC-12 score measured during the first round was 5.40 (SD: 3.20) points and 4.20 (SD: 3.40) in the second round. In the first round, 76% of the participants were classified as having allodynia, while it was 64% in the second round. The reliability of the total ASC-12 score was considered excellent with an ICC of 0.85 (95%CI: 0.64 to 0.93,* p*< 0.0001). The observed ICC for the thermic allodynia dimension of the questionnaire was 0.77 (95%CI: 0.52 to 0.88,* p*< 0.0001), 0.86 (95%CI: 0.75 to 0.92,* p*< 0.0001) for static mechanical allodynia dimension and 0.84 (95%CI: 0.68 to 0.91,* p*< 0.0001) for dynamic mechanical allodynia. The German version of the ASC-12 presented a SEM of 1.15 points and a SDC of 3.40 points.

The internal consistency measured by Cronbach’s alpha was considered adequate since 0.70 was observed for the total score, 0.70 for the thermic allodynia dimension, 0.70 for static mechanical allodynia dimension and 0.80 for dynamic mechanical allodynia dimension.

#### Construct validity

Weak to moderate positive correlations were found between the total scores of the ASC-12 and headache intensity (r= 0.38, *p*= .004), headache disability (r= 0.37, *p*= 0.004) and cold pain thresholds (r= 0.28, *p*= 0.025). The thermic allodynia dimension of the ASC-12 was moderately correlated with headache disability (r= 0.36, *p*= 0.005) and with both cold (r= 0.36, *p*= 0.005) and heat pain (r= -0.30, *p*= 0.010) thresholds. The static mechanical allodynia scores of the ASC-12 presented weak and positive correlations with mechanical pain threshold (r= 0.29, *p*= 0.019) and with mechanical pain sensitivity for all pin-prick stimulators (r values between 0.24 and 0.28, *p*< 0.045). Dynamic mechanical allodynia scores of the ASC-12 were positively correlated with headache intensity (r= 0.37, *p*= 0.004) and headache disability (r= 0.29, *p*= 0.023). These results are shown in Table 3.

**Table 3:** Correlations between clinical and QST outcomes with the German version of the Allodynia Symptom Checklist (ASC-12) total scores and respective dimensions

	Total score	Thermic allodynia	Static mechanical allodynia	Dynamic mechanical allodynia
Headache onset	-0.05	-0.03	-0.03	-0.10
Headache frequency	-0.08	-0.12	-0.06	0.00
Headache intensity	0.38**	0.05	0.08	0.37**
HIT-6	0.37**	0.36**	0.15	0.29*
Cold pain threshold	0.28*	0.36**		
Heat pain threshold	-0.18	-0.30**		
Mechanical pain threshold	0.02		0.29*	-0.05
Mechanical pain sensitivity (8)	-0.04		0.26*	-0.10
Mechanical pain sensitivity (16)	-0.04		0.26*	-0.09
Mechanical pain sensitivity (32)	-0.07		0.24*	-0.11
Mechanical pain sensitivity (64)	-0.08		0.25*	-0.13
Mechanical pain sensitivity (128)	-0.07		0.25*	-0.11
Mechanical pain sensitivity (256)	-0.07		0.24*	-0.10
Mechanical pain sensitivity (512)	-0.06		0.24*	-0.09
Mechanical pain sensitivity (CW)	-0.03		0.27*	-0.06
Mechanical pain sensitivity (QT)	-0.01		0.28*	-0.04
Mechanical pain sensitivity (BR)	-0.02		0.27*	-0.04

*QST* Quantitative sensory testing, *HIT-6* Headache impact test, *CW* Cotton wisp, *QT* Q‐tip, *BR* Brush

^*^p< 0.05, ** *p*< 0.01

#### Agreement analysis

**Fig. 2:**
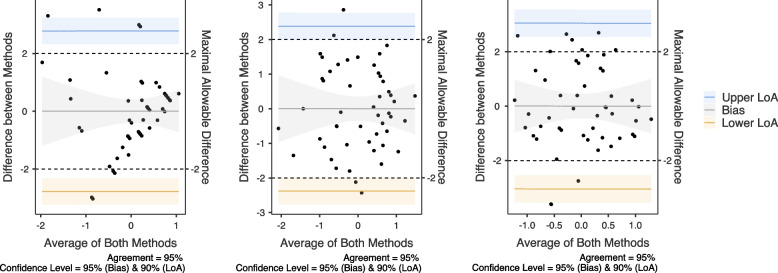
Bland-Altman plots exhibiting the agreement between the Z-scores of the ASC-12 and mechanical pain thresholds (**A**), cold pain thresholds (**B**) and heat pain thresholds (**C**)

The LoA between the ASC-12 and *mechanical pain thresholds* normalized scores (A, Fig. 2) ranged from -2.8 (90%CI: -3.2 to -2.3) to 2.8 (90%CI: 2.3 to 3.2) with a non-significant bias of 0.03 (95%CI: -0.4 to 0.4). The 90%CI of the agreement hypothesis test was between -3.2 to 3.2, *p*> 0.05. The LoA between the ASC-12 and *cold pain thresholds* normalized scores (B, Fig. 2) ranged from -2.4 (90%CI: -2.8 to -2.0) to 2.4 (90%CI: 2.0 to 2.8) with a non-significant bias of 0.02 (95%CI: -0.3 bis 0.3). The 90%CI of the agreement hypothesis test was between -2.7 to 2.7, *p*> 0.05. The LoA between the ASC-12 and *heat pain thresholds* normalized scores (C, Fig. 2) ranged from -3.0 (90%CI: -3.5 to -2.5) to 3.1 (90%CI: 2.6 to 3.6) with a non-significant bias of 0.02 (95%CI: -0.4 bis 0.4). The 90%CI of the agreement hypothesis test was between -3.5 to 3.5, *p*> 0.05. Although almost all individuals were within the 95% limits of agreement range of the Bland-Altman plots for all three analysis and no systematic bias was observed, the agreement hypothesis test was rejected, indicating overall disagreement between ASC-12 and mechanical, cold and heat pain thresholds.

## Discussion

In this study, we successfully translated and adapted the English version of the ASC-12 to German, considering semantic and idiomatic equivalence to the original version. In addition, the German version of the ASC-12 demonstrated adequate measurement proprieties, despite the lack of agreement with thermal and mechanic thresholds.

For the cross-cultural comparison between different languages, the translation process has to be rigorous to ensure the equivalence of versions, and has to include assessments of the translation quality by an expert committee [27]. To this date, the ASC-12 was available only in English [3], Portuguese [28] and Turkish [29]. The German and the Portuguese versions of the ASC-12 were developed based on the same translation and cross-cultural guideline described by Beaton et al [16]. Nonetheless, a standardized process does not ensure equivalent measurement proprieties to the original version. For this reason, the assessment of validity and reliability of the adapted version are still required [30].

The structural validity analysis of the German ASC-12 confirmed the same original three questionnaire domains [3], as also observed in the Turkish version [29]. The items “*combing your hair*” and “*pulling your hair back*” demonstrated the highest contribution to the model fit, similar to Lipton et al [3]. On the other hand, and similar to the Turkish version [29], the items “*wearing contact lenses*” and “*shaving your face*” did not contribute significantly to the questionnaire, probably due to the low number of responses in the sample. Ashkenazi *et al.* also suggested that the item “*shaving your face*” does not add value in the identification of patients with allodynia [31]. This can be attributed to the higher prevalence of migraine among women, similar to the non-frequent use of contact lenses, especially during a migraine attack. Interestingly, the avoidance behavior towards some items of the ASC-12 during the migraine attack was confirmed by some patients during the translation and cross-cultural process of the questionnaire. This can lead to a reduction in the detection of allodynia, since patients reported not having experienced the activity and rated “*does not apply to me*”, despite anticipated discomfort and hence avoidance of the activity during a migraine attack.

Similar to the Turkish [29] and Portuguese [28] versions of the ASC-12, an adequate internal consistency and reliability was observed in the German version. Furthermore, the prevalence of allodynia verified in the sample is in line with previous reports [1, 3, 28, 29]. For the first time, measurement error variables of the questionnaire were calculated, which can inform about the absence of real changes over time and absence of differences between individuals [25]. The current results indicate that differences of scores lower than 1.15 points should be interpreted as an error, as well as score changes lower than 3.4 points. These results are relevant for all researchers and clinicians that may use the ASC-12, since it is crucial for an adequate interpretation of questionnaire scores. Future studies should also consider performing responsiveness analysis in order to determine minimal important change values of the ASC-12, which would indicate presence or absence of clinically relevant changes of ASC-12 scores in both future clinical trials as well as in clinical practice.

Further than adapting the ASC-12 to German, this is the first study that performed the construct validity and absolute agreement analysis in relation to the gold standard of measurement, the QST [14]. A previous study evaluated the presence of allodynia in migraineurs using the ASC-12 and a thermal stimulator, measuring heat and cold detection thresholds before and after onabotulinumtoxinA injection [32]. The authors reported improvement of ASC-12 scores after the treatment but no changes in thermal thresholds, further than no correlation between the questionnaire and the QST examination [32].

Indeed, the ASC-12 seems to be very accurate to identify patients with allodynia in contrast to the QST protocol, since - despite the questionnaire recall bias - it refers to symptoms occurring during a migraine attack [31]. Meanwhile, the QST protocol is usually performed during the interictal phase of migraine [33]. Although migraineurs often report several interictal symptoms [34], the level of hypersensitivity more likely returns to baseline in episodic patients during the headache-free period [35, 36]. In our measurement properties sample, 42% of the patients reported having a migraine attack in the previous or following 48hs of the examination. Among other factors, this may have contributed to the significant correlations between the thermal and mechanical domains of the ASC-12 with the thermal and mechanical pain thresholds, confirming the construct validity of the questionnaire. However, the absolute agreement analysis indicated that despite the absence of a significant bias, the QST and ASC-12 measures tended to disagree. It suggests that the QST cannot be replaced by the ASC-12 evaluation in research and clinical practice. Future studies may demonstrate agreement and greater correlation values by considering the same migraine phase for both assessments, i.e. performing the QST protocol during the headache phase and/or including a bigger sample of chronic migraine patients.

Further than the associations between thermic and mechanical aspects of the ASC-12 and QST, these current data showed correlations between the total scores of the ASC-12 with headache intensity, disability levels and cold pain thresholds. The association between greater migraine severity and allodynia was already demonstrated in previous reports [1, 3, 7, 29, 37]. Han et al [37] demonstrated in a populational study that the presence of allodynia was associated with higher headache frequency, headache intensity, presence of anxiety and depression. These current findings also agree with the study of Seo & Park, which showed among other factors, higher disability and headache intensity levels in migraineurs with allodynia and sensory hypersensitivity [7]. Burstein *et al.* reported greater changes on cold – but not heat – pain thresholds one hour after the onset of a migraine attack [38]. This suggests that cold threshold is the first modality to demonstrate abnormalities and identify allodynia in patients with migraine.

The advances in the understanding and evaluation of allodynia in patients with migraine can open perspectives and impact positively on new strategies of treatment. Recent literature links allodynia to dysfunctional analgesic mechanisms and abnormal representation of pain perception [39], which are associated with migraine chronification [40, 41] and observed more prominent during migraine attacks [42]. Interestingly, the presence of interictal allodynia can identify with 85% of accuracy non-responders of CGRP monoclonal antibodies [43]. The skin hypersensitivity can be explained by a hypofunction of the descending pain inhibitory system, which modulates the nociceptive transmission, or by a facilitation of the ascending pain system [44]. Neuroimaging studies suggest that patients with allodynia identified using the ASC-12 questionnaire demonstrate greater activation of the middle frontal gyrus, somatosensory and anterior cingulate cortex [39], along with abnormal sensorimotor connectivity [45, 46]. Despite the advances in the understanding of the hypersensitivity in migraineurs, they do not yet provide a clue to the prevention and treatment of allodynia and its influence on the course and burden of the disease. Further investigation of this phenomenon and its pathophysiology can open possibilities to address allodynia within the treatment plan, with the aim to reduce the likelihood of progression to chronic migraine in this group of patients. Since the ASC-12 can be a valuable tool not just for clinical but also neuroimaging and basic research, it is important to understand its measurement proprieties, its limitations and move towards an improvement of the tool, especially in regard to agreement with the gold standard.

It is important to highlight some limitations of the present study. The study sample was composed mainly by female patients with episodic migraine, which were assessed in a headache-free period. The predominance of females could have influenced the low contribution of the item “*shaving your face*” to the identification of allodynia by the questionnaire. The assessment of patients in the headache-free period and low proportion of chronic migraine patients in the sample are likely to limit the interpretation of the correlation and agreement analysis. Despite these limitations, the German version of the ASC-12 questionnaire presented similar measurement proprieties to the original in English, Portuguese and English versions, being available for both research and clinical settings in German-speaking countries.

## Conclusions

The German version of the ASC-12 provides a valid and reliable tool for evaluating the presence and severity of cutaneous allodynia among German speaking patients with migraine. Despite the correlation between the ASC-12 questionnaire and the QST evaluation, one method cannot be replaced by the other in the evaluation of cutaneous allodynia.

### Supplementary Information

**Additional file 1.**

## Data Availability

The datasets used and/or analyzed during the current study are available from the corresponding author on reasonable request.

## Abbreviations

ASC-12: Allodynia Symptom Checklist
BR: Brush
CFI: Comparative fit index
CW: Cotton Wisp
HIT-6: Headache Impact Test Questionnaire
ICC: Intraclass Correlation Coefficient
LOA: Limits of agreements
RMSEA: Mean Square Error Root of Approximation
NRS: Numeric Pain Scale
QT: Q‐tip
QST: Quantitative Sensory Testing
SDC: Smallest Detectable Change
SEM: Standard Error of Measurement
SRMR: Standardized Root Mean Square Residual
TCS: Thermal Contact Stimulator
TLI: Tucker-Lewis-Index
