# Supplementary material for: Translation, cross-cultural adaptation and measurement proprieties of the German version of the Allodynia Symptom Checklist (ASC-12)
Source: J Headache Pain. 2023 Dec 1;24(1):160. doi: 10.1186/s10194-023-01697-9 (PMC10693116; doi:10.1186/s10194-023-01697-9)
Supplement: Supplementary file 1 — Additional file 1. [file 10194_2023_1697_MOESM1_ESM.pdf]

12-item Allodynie Symptom Checkliste (ASC-12) – German version

Wie oft haben Sie während Ihrer stärksten Kopfschmerzen eine unangenehme Hautempfindung oder verstärkte Schmerzen bei folgenden Handlungen?

	**betrifft mich nicht^{0}**	**Niemals^{0}**	**Selten^{0}**	**in weniger als der Hälfte der Zeit^{1}**	**in der Hälfte der Zeit oder mehr^{2}**
beim Haare kämmen					
beim Zurückhalten der Haare (z. B. Pferdeschwanz)					
beim Gesicht rasieren					
beim Tragen von Brillen					
beim Tragen von Kontaktlinsen					
beim Tragen von Ohrringen					
beim Tragen einer Halskette					
beim Tragen enger Kleidung					
beim Duschen (wenn das Duschwasser auf Ihr Gesicht prasselt)					
beim Ablegen des Gesichts oder Kopfes auf einem Kissen					
bei Hitze (z.B. Kochen, Waschen des Gesichts mit heißem Wasser)					
bei Kälte (z.B. mit einem Kühlpacks, Waschen des Gesichts mit kaltem Wasser)					
**Summe:**					
**Gesamtsumme:**					

Allodynie	Summe
keine	0-2
leichte	3-5
moderate	6-8
schwere	9 oder mehr
